# An increase in ALS incidence on the Kii Peninsula, 1960-2009: A possible link to change in drinking water source

**DOI:** 10.3109/17482968.2012.674140

**Published:** 2012-05-28

**Authors:** Tameko Kihira, Sohei Yoshida, Tetsuya Kondo, Keiko Iwai, Sachiko Wada, Satomi Morinaga, Yoshinori Kazimoto, Tomoyoshi Kondo, Kazusi Okamoto, Yasumasa Kokubo, Shigeki Kuzuhara

**Affiliations:** 1 Department of Health Sciences, Kansai University of Health Sciences, Kumatori-cho, Sennan-gun, Osaka; 2 Faculty of Nursing, Kansai University of Health Sciences, Kimiidera,Wakayama-city,Wakayama; 3 Department of Neurology, Wakayama Medical University, Kimiidera,Wakayama-city,Wakayama; 4 Department of Public Health, Aichi Prefectural College of Nursing and Health, Nagoya; 5 Department of Neurology, Mie University Graduate School of Medicine, Tsu-city, Mie; 6 Department of Medical Welfare, Suzuka University of Medical Science, Suzuka, Mie, Japan

**Keywords:** Focus area, Kii-ALS, incidence, new cluster

## Abstract

We investigated changes in the incidence of amyotrophic lateral sclerosis (ALS) in the Koza/Kozagawa/Kushimoto area (K. area) in the Kii Peninsula, Japan in 1960–2009. Probable and definite ALS cases diagnosed using El Escorial criteria were collected during a five-decade period: period I-V, 1960–2009. Forty-three ALS patients matched the selection criteria in the overall K. area, including three patients on Oshima, a small island opposite the mainland K. area. The age- and gender-adjusted incidence of ALS in the overall K. area (standardized for the 2005 Japanese population) decreased from 5.47/100,000 (95% CI 1.86–9.08) in period I to 0.61/100,000 (95% CI-0.28–1.50) in period **III**, and then increased to 4.39/100,000 (95% CI 1.70–7.07) in periodV. On Oshima, the age- and gender-adjusted incidence of ALS was 9.45/100,000 (95% CI—7.39–26.29) in period V. The present research indicates an increase of ALS incidence in the K. area, especially on Oshima. A limitation of this study was the small population.

## Introduction

Amyotrophic lateral sclerosis (ALS) is a devastating adult-onset degenerating disease of unknown etiology of the motor neuron systems. The Koza, Kozagawa and Kushimoto (K.) area in the Kii Peninsula of Japan was reported to have a higher incidence of ALS in the 1950s than other areas of the world (1–5). Epidemio-logic research showed that drinking water sourced from Kozagawa River in the K. area contained severely low levels of Ca and Mg, and Ca/Mg deficiency was speculated to have a role in the development of ALS in these areas (5,6). On Oshima, a small island municipally included in the K. area, the source of drinking water was changed from regional water to the Kozagawa River in 1975. To clarify whether ALS epidemiology on Oshima changed after altering the water source, we investigated changes in ALS incidence on Oshima and in the K. area in 1960–2009.

## Methods

### Area of investigation

**Figure 1:**
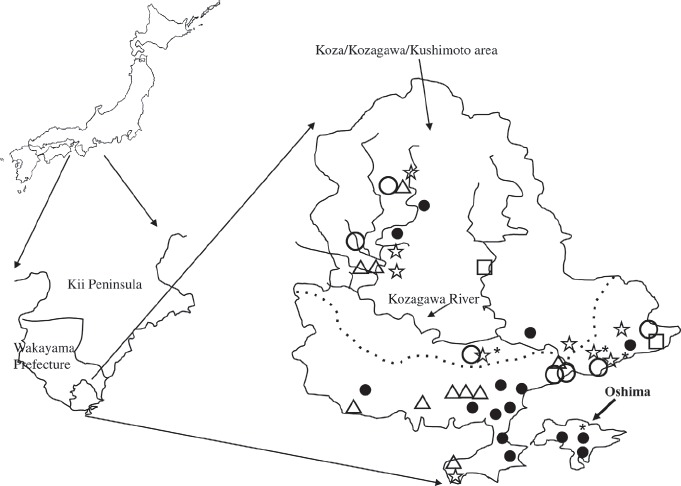
Geography of Koza/Kozagawa/Kushimoto area and Oshima, and distribution of patients with ALS. Patients with ALS between 1960 and 1969 (period I: º), between 1970 and 1979 (period II: Δ): between 1980 and 1989 (period III: □), between 1990 and 1999 (period IV: ☆), and between 2000 and 2009 (period V: •) were plotted. *: ALS/PDC

The K. area (population: 23,357 in 2005 census, 430.3 km^2^) is located in the southern part of Wakayama Prefecture of the Kii peninsula, Japan (Figure 1). Oshima (population: 1279 in 2005 census, 9.93 km^2^) is an island opposite the K. area. The K. area has a local public healthcare and welfare center, two local public hospitals, two private hospitals and 19 medical clinics.

### Incidence of ALS in 1960–2009

ALS patients on Oshima and overall in the K. area were enrolled from multiple sources, including hospital medical records and reports from local medical staff over a five-decade period: period I, 1960–1969; period II, 1970–1979; period III, 1980–1989; period IV, 1990–1999; period V, 2000–2009. Regional physicians including neurologists in hospitals and clinics and the staff of the public healthcare and welfare center in the K. area were requested by our team to report all patients with possible motor neuron disease every year (7). To ensure complete case identification, Wakayama Prefecture's List of Patients with Intractable Disease certified by the Ministry of Health and Welfare of Japan was used. The selection criteria were as follows: 1) patients who our neurologists examined and diagnosed using the El Esco-rial criteria (8); 2) patients with probable or definite ALS who had been living in the K. area, including Oshima, for at least one year before diagnosis. Patients who showed Parkinsonism or dementia during the disease course (ALS/Parkinsonism dementia complex: ALS/PDC) were also included.

The present research was approved by the ethics committee of Wakayama Medical University and Kansai University of Health Sciences.

### Statistics

A direct method was used to standardize the annual incidence rates by age and gender, using populations in the 2005 census in Japan: 127,537,189. Unpaired t-test was performed and two-sided p<0.05 was considered significant.

## Results

### Patient population and clinical characteristics

We enrolled 50 patients with definite or probable ALS in the K. area, including Oshima, in 1960–2009, and 43 patients matched the selection criteria (Table I) (seven were excluded because they were living outside the K. area when diagnosed). Patient distribution was restricted to the mainland side of the K. area during periods I-IV. Three ALS patients were found on Oshima (two males and one female) in period V (Figure 1).

**Table I:** Patients with ALS in the K. area, including Oshima in each period.

	Population&			ALS patients matched for the selection criteria				
Period	Overall K area	Oshima	Total ALS patients enrolled (»)	Overall K area	Oshima	Age at onset Mean (S.D.)	M/F ratio	FH *n* (%)
I	18,251#	2,823	7	7	0	56.1 (10.8)	2.5	0(0)
II	32,128	2,095	12	10	0	55.3 (10.6)	1.5	1 (10)
III	29,732	1,756	4	2	0	59.0 (7.1)	2.0	0(0)
IV	26,405	1,508	11	9	0	57.7 (11.5)	2.0	3 (33)
V	23,357	1,279	16	15	3	67.6 (12.3)*	0.88	2(13)
Total			50	43	3		1.53	6 (14.0)

K.: Koza/Kozagawa/Kushimoto area; M/F: male/female ratio; FH: familial history.

& : populations in 1965, 1975, 1985, 1995 and 2005 census.

# : Koza/Kozagawa area.

* : £<0.01 when compared with the mean age at onset in period I.

The mean age at onset in period V was the highest among the periods (Table I). The male: female ratio was low in period V compared to periods I-IV. The frequency of cases with a positive familial history in a detailed interview was 14.0%. Cu/Zn super-oxide dismutase (SOD1) genes were analyzed in three of six familial cases; none had a SOD1 gene mutation. Three ALS/PDC patients were found in the K. area in period IV and one patient on Oshima in period V.

### Incidence of ALS in 1960–2009

**Figure 2:**
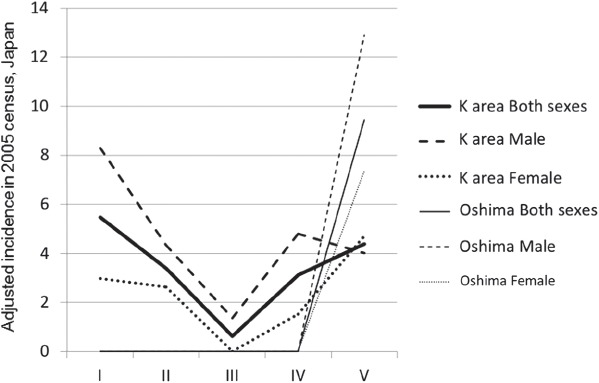
Changes in incidence of ALS in the overall Koza/Kozagawa/Kushimoto (K.) area and Oshima between 1960 and 2009. Comparing the incidence by the periods, the age- and gender-adjusted incidence of ALS in the overall K. area for the 2005 Japanese population was 5.47/100,000 (95% CI 1.86–9.08), male: 8.29/100,000 (95% CI 1.84–14.73), female: 2.98/100,000 (95% CI-0.69–6.66) in period I, markedly decreased to 0.61/100,000 (95% CI-0.28–1.50), male: 1.36/100,000 (95% CI-0.58–3.30), female: 0 in period III, but recently increased again to 4.39/100,000 (95% CI 1.70–7.07), male: 4.01/100,000 (95% CI 0.22–7.81), female: 4.71/100,000 (95% CI 0.93–8.49) in period V.

The mean annual crude ALS incidence in the K. area in period V was 6.42/100,000 and that on Oshima was 23.46/100,000. The age- and gender-adjusted ALS incidence in the K. area decreased from 5.47/100,000 (95% CI 1.86–9.08) in period I to 0.61/100,000 (95% CI-0.28–1.50) in period III and then increased to 4.39/100,000 (95% CI 1.70–7.07) in period V (Figure 2). On Oshima, the age-and gender-adjusted incidence of ALS was 9.45/100,000 (95% CI-7.39–26.29) in period V.

## Discussion

The age- and gender-adjusted ALS incidence in the K. area decreased from 5.47 in 1960–1969 to 0.61 in 1980–1989, and then increased to 4.39 in 2000–2009. The declining trend of the male: female ratio in the past 10 years was comparable with other reports (9,10), and the recent increase of ALS incidence in females could be related to some type of environmental or cultural factor pertaining in females in this area and the increased confirmation of older female patients (11). The reason for the decline in 1980-1989 is not clear, but might be partially due to missing cases from the data set, emigration, and environmental, lifestyle and cultural changes. Recent reports of annual age-adjusted ALS incidences ranged from 0.42/100,000 (12) to 2.96/100,000 (13) in other areas in the world (14–17). Taken together, the age-and gender-adjusted incidence in the K. area and Oshima in 2000-2009 was higher than in other areas. On Oshima, no patient with ALS was found in previous research in 1946–1965 (18). It is noteworthy that a high ALS incidence was first found on Oshima in 2000–2009 after the drinking water source was changed to the Kozagawa River in 1975. The present research indicated an increase of ALS incidence in the K. area, especially on Oshima. A limitation of this study was the small population. Continuous study over a longer period is needed in this area.
